# How did the COVID-19 pandemic impact the stress vulnerability of employed and non-employed nursing students in Romania?

**DOI:** 10.1371/journal.pone.0264920

**Published:** 2022-03-04

**Authors:** Mihaela Simionescu, Elena-Nicoleta Bordea, Angelo Pellegrini

**Affiliations:** 1 Institute for Economic Forecasting, Romanian Academy, Bucharest, Romania; 2 Faculty of Midwifery and Nursing, "Carol Davila" University of Medicine and Pharmacy, Bucharest, Romania; St John’s University, UNITED KINGDOM

## Abstract

In the light of the current COVID-19 pandemic, being considered a present challenge for public health, the main purpose of this work is to analyze the vulnerability to stress of a sample of nursing students in Romania considering their status on labour market (employed students in the medical system and non-employed students) before and during the COVID-19 pandemic. Employed students were more vulnerable to stress comparing to non-employed ones during the pandemic. In addition to this, the nursing students working in the medical system experienced medium vulnerability to stress during the pandemic comparing to those working before the pandemic who experienced a low vulnerability to stress. Excepting the non-employed students before the pandemic, the females were more vulnerable to stress comparing to the males in the sample and the students living in the country experienced a higher level of stress comparing to those living in the urban area. During the pandemic, most of the employed nursing students expressed their fear of getting infected with COVID-19, this representing the most stressful factor for them, while most of them mentioned the self-control as being the most proper strategy for them to cope with stress. These empirical findings have practical implications for stress control among present and future nurses, for management of medical units and for higher education nursing.

## Introduction

The COVID-19 pandemic is considered a major challenge for public health. In this context, nursing students preparing themselves for this work should be key factors in the struggle against the virus infection, prevention and control. The experience of nursing students with medium studies in this field and who already work in the medical system is essential for their colleagues who are not employed as nurses yet. However, the studies related to stress among nurses and nursing students are limited during the COVID-19 pandemic, because a survey is considered to place undue stress on an individual. Good mental health for actual and future nurses is vital in order to control different infectious disease [1]. Most of the studies have been related to the epidemiological aspects of the pandemic, but only several works have analyzed the factors affecting nurses’ stress during the pandemic [2–4].

The Romanian medical system has been severely affected by the COVID-19 pandemic and this new challenge requires nurses to become more than healthcare suppliers and to act like architects of the new health system. Work stress management should be the cornerstone of the new system and this should start earlier with better education for nursing students who prepare themselves to face work challenges. In Romania, nursing students with medium studies in this field (sanitary high school and sanitary post-secodary school) have the right to work as nurses in public and private medical units in any medical specialization. Superior studies in this field are not compulsory and do not depend on medium studies in specialized education centers. They provide additional advantages for nurses: higher salaries comparing to nurses with medium studies and the right to be promoted as head of department in a medical unit. The superior education for nursing students in Romania should be improved by supporting them in managing the stressful work situations. From this point of view, our study is a novelty for the specialized literature in Romania, because the vulnerability to stress of nursing students is analyzed before and during the COVID-19 pandemic and some strategies are suggested during the pandemic in order to cope with stress.

Most of the studies from literature have analyzed the factors of stress for nurses utilizing a qualitative approach, but our research presents a quantitative approach. Comparing to previous studies, two perspectives on nursing students are considered: employed nursing students before and during the COVID-19 pandemic and nursing students who are not employed yet. The difficult context of the actual pandemic constraint students preparing themselves to become nurses and other recommendations should be made in order to surpass this constraint. Therefore, the subjects of this study are represented by nursing students: employed nursing students working in the medical system and non-employed nursing students. The employed nursing students working outside the health sector are not considered in this analysis.

One of the challenges of human resources is work stress that diminishes work efficiency and causes negative effects for people’s souls and bodies [5]. Work stress is defined as the emotional and physical consequences of the interaction between human resources and working environment or the lack of a workplace and the ability to respond to these demands [6]. In other words, work’s duties are too demanding comparing to the individual’s social, emotional, physical, psychological or economic resources [7].

Nursing is considered a stressful work which impacts the physical and mental health of the nurses, but it also causes negative effects on the health system, especially for patients [8]. The US Occupational Safety and Health Institute has declared that nursing work is ranked as the 27th in a list of 130 professions that could be associated with mental health issues [9]. In addition, nursing involves a higher level of stress comparing to other medical positions. A study conducted in India has indicated 87.4% cases of nurses reporting stress, while, in Saudi Arabia, the percent has been lower (45.5%). Average and high work stress has been noticed in the case of 57.4% nurses from Iran [10].

Different categories of stressors have been identified in previous studies: a lot of pressure at work [6], violent acts and night shift work [7], patients with unsecure conditions and emergency cases [5], conflicts with colleagues and poor teamwork [6]. During the COVID-19 pandemic, several studies have been conducted in order to identify the stress factors for nurses. For example, in China, the stress factors in the case of nurses have been the following: anxiety, working hours per week and child-care, a multiple regression being estimated [3]. During the COVID-19 pandemic, a survey conducted among 662 nursing students from Turkey has indicated a moderate level of stress that is correlated with gender, age and aspects related to the COVID-19 pandemic. Unlike this study, in our research we consider the difference between employed nursing students in the medical system and non-employed ones. The purpose is to identify differences between the two categories and indicate the best strategies to cope with stress in case of employed nursing students who have already worked in the medical system.

The effects of stress consist of: difficult decision to make, anxiety, less motivation for this work, apathy, decrease in the quality of life, risk of chronic diseases, less immunity [11].

The negative effects of work stress for nurses impose recommendations for coping with stress. People who are successful in solving their problems, have coped with stress in a better manner comparing to those with a defensive strategy [12]. In this context, nurses have employed a variety of coping mechanisms.

There are more fields that have provided contributions to stress theory (sociology, psychology, psychiatry, psychobiology and anthropology). Our approach is focused on psychiatric theory. Life events, the COVID-19 pandemic being an example, are considered as factors of stress that change the people’s behavior and the long-run pattern [13]. In our case, the epidemic has made people more cautious and more interested in maintaining the hygiene rules, which is an additional stressor, because it is more time consuming. The stress is higher for the students working in the medical system, because the possibilities of getting infected are higher and the virus could be transmitted to family members. The stage theory of crisis developed by Caplan could explain people’s attitude regarding the pandemic [14]. Higher stress represents a protection measure that makes students to engage more in maintaining the rules in order to reduce the infection risk. From this point of view, stress represents a step forward in their personal development.

Several directions of research have been recently identified in the context of work motivation during the COVID-19 pandemic. First of all, burnout has increased during the COVID-19 pandemic because of isolation [15, 16], understaffed and burden of work [17]. The Romanian universities have provided online courses since the beginning of the COVID-19 pandemic, which have the following implications for our research: a) non-employed nursing students experienced less stress because of fewer risks to get infected due to staying home comparing to their colleagues working as nurses; b) non-employed nursing students were more vulnerable to stress during the pandemic comparing to the pre-epidemic period: c) employed nursing students experienced more stress comparing to non-employed ones because they have to attend online courses and to work more; d) employed nursing students were more vulnerable to stress during this medical crisis comparing to the pre-epidemic period because of understaffed and overwork. One of these ideas is also supported by Schwartzman who has showed a higher stress in case of employed students comparing to non-employed ones during the pandemic [18]. In addition, online teaching is less preferred by students than face-to-face teaching because there are no interactive practices and activities and because of the problems with Internet connection [19]. Third of all, workplace motivation might change during the pandemic [15]. In our case, both employed and unemployed students might decide to work in another field, at least on a temporary basis, in order to minimize pandemic challenges, including fear of getting infected and burnout.

More hypotheses have been formulated in order to validate them on empirical data. For example, employed students would not have had a higher stress vulnerability than non-employed ones before the pandemic because of their income and the respect earned owing to their chosen profession. Prior to the pandemic, non-employed students may have been exposed to stress related to not having employment in their field comparing to employed students.

Many theories have explained the low rates of psychological comfort for non-employed people, the most known one being the Jahoda Deprivation Model. This model shows that being employed satisfies the basic needs of individuals: financial needs and psychological ones (social interaction, implication in traditional activities that help the others, common efforts in order to achieve specific objectives) [19]. Unemployed people are more exposed to deprivation in terms of financial resources and psychological needs. Students not working are less exposed to deprivation than unemployed people who are not included in educational programs due to interaction with colleagues and professors. However, this interaction does not provide non-employed students a sense of usefulness as a workplace does. Deprivation of psychological needs is considered a cause of stress in the case of non-employed students [15]. Another explanation is related to the fact that people should be active members of society and a workplace helps them in this respect [19]. People’s earnings help them organize their life in a more pleasant way and that is not always possible for individuals without a workplace. In this case, the failure to reach aspirations is because of the poor financial resources and that represents an additional cause of stress.

On the other hand, the level of stress for employed students might be higher during the epidemic, comparing to non-employed ones because of the infection danger. According to the conversation of resource theory, work stress is caused by the threats of working environment [20]. The COVID-19 is an additional environmental threat to nursing students working in the medical system, while the non-employed students are less exposed to this source of risk. On the other hand, the lack of enough financial resources might be a stressor for non-employed nursing students, but this medical crisis helps them to find employment faster in the health sector. In Romania, the high demand for medical personnel provides good perspectives for people who want to follow a career in this field.

The theoretical description of the stress factors and strategies in order to overcome this, should be followed by an empirical analysis [21]. In this work, following the description of the methodology, the empirical analysis is conducted based on a sample of nursing students from Bucharest, Romania, the purpose being to compare the vulnerability to stress and to identify the specific stress factors and strategies to cope with stress, concerning employed nursing students during the pandemic. The results are widely discussed, including practical implications and limitations of the research. The last part of this work presents the main results and future directions of research.

## Methodology

This analysis is focused on graduate nursing students from the Faculty of Midwifery and Nursing from Bucharest, the single faculty of this kind in the city. Two different samples of students have been considered in this analysis: a sample of students who responded to the survey before the COVID-19 pandemic (June 2019) and a sample of students who responded to the survey during the pandemic (June 2020). The oral consent has been obtained from students and from the rector of the university. The approval of the ethics committee of the faculty was not necessary in this case since the study was based on students who had to complete an opinion survey without treating them as patients. The students expressed their volition to complete the survey without any constraint.

Employed students are those who have a workplace. In this study, we have included only those employed nursing students working in the medical system as nurses in public and/or private hospitals, dispensaries or clinics and non-employed nursing students. Since the information is confidential, the employed individuals have not been asked to write the name of their employer. There are some students not working in the healthcare industry, but these ones have not been included in our sample. Since their number is low, a comparison with those working in the health sector would not be relevant.

The total number of students was 270 in June 2019, but only 268 of them were employed as nurses or non-employed and they responded to the survey. For the period of the pandemic (June 2020), another 268 students were selected out of a total of 270 students (employed as nurses and non-employed). However, the samples are not representative at national level according to different characteristics, but they are relevant for nursing students in Bucharest, the capital of Romania. An online voluntary survey was utilized to collect data. The link of this survey was sent to nursing students by email.

Miller and Smith scale has been used to assess the vulnerability to stress based on 20 questions in the survey concerning psychological features (introverted or extroverted individual), social and behavioural factors (income, religion, social support, smoking, lifestyle, energy drinks, and excessive alcohol consumption), and biological factors (weight, health status) [22]. Miller and Smith scale has been selected in this study since it is easy to apply in psychological and psychiatric research for all categories of individuals regardless their level of vulnerability to stress. The presentation of the questions in the survey and the details about the calculation of the scores used to establish the categories of vulnerabilities to stress are made in the Appendix 1 in S1 File. The first 20 questions are properly used to calculate the scores. Two additional questions were added in the survey in order to identify the most stressful factor and the most efficient coping strategy for employed nursing students only during the pandemic. Scores have not been assigned to these questions, because these are not used to assess vulnerability to stress.

The stressors refer to: fear of getting sick with the new coronavirus, lack of experience in managing a pandemic, miscommunication and social distance, more patients comparing to the pre-pandemic period, mask wearing, the use of new equipment purchased during the pandemic, huge workload, more frequent use of disinfectants.

Several coping strategies at work have been indicated since the beginning of the pandemic and the respondents have been asked to choose the most efficient one for them: self-control, colleagues’ support, implication of medical unit management, family’s support and spiritual dimension reflected from various perspectives like contact with nature, recognision of God’s power, use of prayers and holy books, spiritual improvement.

The Alpha (Cronbach) coefficient has been computed for checking the internal consistency among the scale’s items. The calculated value of Cronbach’s Alpha coefficient is 0.76, suggesting good consistency.

Apart from these questions, the students have been asked to provide answers in order to register some demographic characteristics:

- Gender (female/male);
- Marital status (married/unmarried);
- Age group (18–25 years, 26–29 years, 30–39 years, 40–49 years, 50–65 years);
- Environment (urban/rural environment);
- Status on labour market (employed/non-employed);
- Living conditions (alone, on rent, with parents, in student dorm).

The statistical analysis is based on: a) treatment effects that estimate experimental-type causal effects using survey data and b) Pearson’s chi-square test to measure the association between vulnerability to stress and various characteristics: demographic variables (gender, age category, marital status, environment) and copying strategies to stress for separate groups of nursing students: employed students before the pandemic, non-employed students before the pandemic, employed nursing students during the pandemic and non-employed ones during the actual medical crisis. In addition, some relative frequencies have been computed in order to identify the most severe stressor concerning the employed nursing students during the pandemic and the most efficient copying strategy to stress in the same period.

In this case, the treatment is influenced by the emerging of the employed nursing students and non-employed ones on the labour market and by the existence of the pandemic period or not (during the pandemic and before the pandemic). In this case, the main advantage of this method is the ability to make comparisons between groups (employed and non-employed nursing students, students before and during the pandemic), while a disadvantage is related to extensive computations. Details on treatment effects method are provided in Appendix 2 in S1 File in the algorithm for assessing stress vulnerability.

Pearson’s chi-square test has been selected as being the most suitable non-parametric test to apply in this study since the hypotheses of the test are fulfilled in this case: a) each two variables for which association is verified are measured at an ordinal or nominal level (category data for demographic variables and copying strategies to stress) and b) the two variables consist of two or more category, independent groups (gender—two groups: males and females), environment (two groups: rural environment and urban environment), age category (five groups: 18–25 years, 26–29 years, 30–39 years, 40–49 years, 50–65 years), copying strategies to stress (five groups: self-control, colleagues’ support, involving of hospital management, family’s support and spiritual dimension).

The Pearson’s chi-square test has the advantage of revealing whether there are significant differences between the groups with respect to a variable. For example, in this case, differences among stress scores are verified concerning males and females, also with regards to people living in villages and those living in urban environment. The main limit of this method is related to inaccurate results for some small samples when the expected frequencies are too low.

Causal effects are defined as comparisons between possible outcomes in terms of expected values, median or odd-rations of an unit *i* and a group of *N* units [23]. Appendix 2 in S1 File describes the algorithm used in order to assess the impact of employment status on nursing students and the COVID-19 pandemic on their stress vulnerability. Two hypotheses are based on the fact that the COVID-19 pandemic is an additional stress factor for both employed and non-employed students. Our hypotheses are supported by a previous study that revealed nurses being more vulnerable to stress during the COVID-19 pandemic comparing to previous period [24]. On the other hand, employed people during the pandemic tend to be more stressed out comparing to non-employed ones [25].

H1a) Employed students do not experience a higher level of stress comparing to non-employed ones before the COVID-19 pandemic.
H1b) Employed students experience a higher level of stress comparing to non-employed during the COVID-19 pandemic.
H2 During COVID-19 pandemic, employed students experience a higher level of stress comparing to employed ones before the pandemic.
H3 During COVID-19 pandemic, non-employed students experience a higher level of stress comparing to non-employed ones before the pandemic.

In addition, some comparisons will be made between employed students before and during the pandemic in terms of coping strategies using Pearson’s chi-square test.

## Empirical analysis

The sample analyzed before the pandemic and the sample that made the subject of the research during the pandemic are separately described. The mean stress score for the sample before the pandemic is 25.55 (standard deviation = 9.96) which suggests low vulnerability to stress. However, during the pandemic, the average stress score reached 27.53 (standard deviation = 8.76) with values ranging from 8 to 60. In this period, 45.2% of the nursing students experienced average and high vulnerability to stress, which required suitable recommendations for stress control. The test used to compare two population proportions indicates no significant differences between the mean stress scores before and during the pandemic (calculated z-statistic = -0.23, p-value = 0.38), but the number of students with high vulnerability to stress increased to 70% during the pandemic comparing to the pre-pandemic period. The stress control is necessary during the medical crisis in Romania in order to avoid the generalisation of cases with high stress.

According to Table 1, the majority of the individuals in both samples is represented by females (82.47% in the sample before the pandemic and 80.6% in the sample during the pandemic). More than 50% of the students in both samples belong to urban environment and more than 70% of them are between 18 and 25 years old in each sample. 80.97% of the respondents in the sample before the pandemic are unmarried, while 75% of them are not married during the period corresponding to the new medical crisis. More than 45% of the students in each sample live with parents. More nursing students were employed during the pandemic comparing to previous period (44.78% comparing to 40.3%). The necessity of more medical personnel during the pandemic and higher salary rewards legally allocated during the state of emergency, led to more nursing students with specialized medium studies in this field, finding a workplace in the medical field. An equal number of students was selected before and during the pandemic in order to respond to the questions of the survey, excluding employed students not working in the medical system: the sample size is 268 students before the pandemic and the sample volume is also 268 students during the COVID-19 pandemic. A number of 178 students responded to this online survey in Romanian, during both periods.

**Table 1:** Samples’ characteristics (before and during the pandemic).

Variable	Relative frequency (sample before the pandemic)	Relative frequency (sample during the pandemic)
**Gender**	17.53% males	19.4% males
	82.47% females	80.6% females
**Environment**	51.11% in urban environment	52.98% in urban environment
	48.93% in rural environment	47.02% in rural environment
**Age group**	18–25 years: 74.62%	18–25 years: 75.74%
	26–29 years: 8.95%	26–29 years: 8.2%
	30–39 years: 6.98%	30–39 years: 6.34%
	40–49 years: 7.09%	40–49 years: 8.98%
	50–65 years: 2.36%	59–65%: 0.74%
**Marital status**	80.97% unmarried	75% unmarried
	19.03% married	25% married
**Living conditions**	45.15% living with parents	48.5% living with parents
	26.86% living alone	25% living alone
	18.28% living in student dorm	15.29% living in student dorm
	9.71% living on rent	11.3% living on rent
**Status on labour market**	59.7% unemployed	55.22% unemployed
	40.3% employed	44.78% employed

Source: personal computations.

In Table 2, the effects of the status of students’ employment regarding the stress scores are evaluated for the entire sample covering the period before the pandemic and during the pandemic, but also during each of these periods. The significance level used in this study is 10%.

**Table 2:** The estimated results of treatment (coping strategies) effects in order to assess the impact of status on labour market regarding stress scores (control variables: Gender, marital status, age group).

Sample	Method	Propensity-score matching	Nearest-neighbour matching	Regression adjustment	Potential outcomes
**Overall sample**	**Coefficient**	5.93*	5.58*	4.72*	16.57*
	**Strong standard error**	2.45	2.56	2.36	1.45
	**Z**	2.42	2.18	1.99	11.44
	**p-value**	0.016	0.029	0.046	0.000
**Before the COVID-19 pandemic**	**Coefficient**	3.74	2.67	1.93	23.80*
	**Strong standard error**	2.62	2.87	2.75	1.76
	**Z**	1.43	0.93	0.70	13.46
	**p-value**	0.153	0.35	0.48	0.00
**During the COVID-19 pandemic**	**Coefficient**	6.21*	6.12*	5.62*	26.61*
	**Strong standard error**	2.27	2.30	2.19	1.40
	**Z**	2.73	2.66	2.56	18.88
	**p-value**	0.006	0.008	0.01	0.000

Note:

* significant at 10% significance level

Source: personal computations in Stata 15.

When the entire sample was analyzed, the unemployed students had an average stress score of 16.57 corresponding to low vulnerability to stress, while the employed nursing students had an average score of around 22 which places them in the same category of stress. However, there is a significant difference between the two groups of students in terms of stress score, since the coefficients associated to all methods are statistically different from 0 to 10% significance level. Corroborating the results, the employed nursing students had a higher average stress score comparing to the students without a workplace throughout the period. This significant difference is explained by the higher scores of employed students during the pandemic comparing to non-employed ones.

Before the COVID-19 pandemic, there were not significant differences in the stress scores of non-employed and employed nursing students. Before the pandemic, the average stress score of students without a workplace was 23.8 (low vulnerability to stress), while the average for employed ones was around 26 (low vulnerability to stress), but the difference is not important, because the coefficients associated to all treatment effects methods are not statistically significant, at 10% significance level.

During the pandemic, the stress scores increased for both categories of students. During this period, the non-employed students have an average score of 26.61 (low vulnerability to stress), while the colleagues working in the medical system have an average score around 33 (medium vulnerability to stress). We can notice that the employed students belong to a superior category of vulnerability to stress during the pandemic, because of the new challenges during this period. According to these results, the hypotheses H1a) and H1b) are verified.

In Table 3, the effects of the COVID-19 pandemic on students stress scores are evaluated for the entire sample, but also for employed nursing students and non-employed students respectively.

**Table 3:** The estimated results of treatment (coping strategies) effects in order to assess the impact of status on labour market regarding stress scores (control variables: Gender, marital status, age group).

Sample	Method	Propensity-score matching	Nearest-neighbour matching	Regression adjustment	Potential outcomes
**Overall sample**	**Coefficient**	4.09*	4.28*	4.17*	23.83*
	**Strong standard error**	1.58	1.67	1.64	1.18
	**Z**	2.58	2.56	2.54	20.05
	**p-value**	0.01	0.01	0.011	0.00
**Employed students**	**Coefficient**	4.42*	4.42*	4.42*	26.17*
	**Strong standard error**	2.69	2.70	2.67	1.88
	**Z**	1.69	1.69	1.66	13.87
	**p-value**	0.09	0.09	0.097	0.00
**Non-employed students**	**Coefficient**	3.56*	2.75	4.04*	22.63*
	**Strong standard error**	1.85	1.81	1.93	1.45
	**Z**	1.92	1.52	2.09	15.54
	**p-value**	0.055	0.13	0.037	0.00

Note: ** significant coefficient at 10% significance level

Source: personal computations in Stata 15.

For the entire sample, the students analyzed during the pandemic had a higher average stress score (around 28) comparing to those who responded before the pandemic and who had an average stress score of 23.83. These average scores correspond to low vulnerability to stress. The difference in the average stress scores of students is significant before and during the pandemic, since the coefficients of all the methods in Table 3 are significant at 10% significance level.

In the group of employed nursing students, the average stress score was around 30 (medium vulnerability to stress) during the pandemic, comparing to the average in the period before the pandemic, when the average score was 26.17 (low vulnerability to stress). The diference in average scores is significant due to statistical coefficients that are different from 0 to 10% significance level concerning all methods.

In the group of non-employed nursing students, the average stress scores was around 26 (low vulnerability to stress) during the pandemic, comparing to an average of 22.63 (low vulnerability to stress) in the period before the COVID-19 pandemic, being lower than the score for employed students. The diference in average scores is significant from statistical point of view due to statistical coefficients that are not null for all methods, at 10% significance level. Therefore, the hypotheses H2 and H3 are verified during the COVID-19 pandemic, comparing to the period before this pandemic.

In addition, we can state that employed nursing students were more vulnerable to stress comparing to their non-employed colleagues, even if both categories were more vulnerable to stress because of the pandemic. The coping strategies for nursing students during the pandemic should be adapted since these are different in some cases from those before the pandemic. Several coping strategies for stress were considered in case of the employed and non-employed students during the pandemic: self-control, spirituality, colleagues’ support, family’s support, manager’s support. In Table 4, the association between stress scores and different variables (gender, environment, age category, marital status, copying strategy for stress) is presented.

**Table 4:** The association between stress scores before and during the COVID-19 pandemic and different variables for employed and non-employed nursing students.

Stress scores for:				
	Employed students before the pandemic	Employed students during the pandemic	Non-employed students before the pandemic	Non-employed students during the pandemic
**Gender**	8.772* (0.000)	8.348* (0.000)	10.236* (0.000)	1.334 (0.248)
**Age category**	1.0147 (0.471)	1.417 (0.233)	1.005 (0.316)	1.893 (0.168)
**Marital status**	1.911 (0.158)	1.893 (0.168)	1.445 (0.229)	1.227 (0.267)
**Environment**	4.578* (0.032)	6.348* (0.011)	7.223* (0.007)	1.817 (0.177)
**Coping strategy for stress**	-	6.003* (0.014)	-	

Source: personal calculations in Stata 15

Note: Values of Perarson’s chi-square statistics in each cell and p-values in brackets

* significant at 10% significance level.

According to Pearson’s chi-square test, there are significant differences between males and females and between students from rural and urban environment regarding their vulnerability to stress in case of employed students before and during the COVID-19 pandemic and in case of non-employed students before the pandemic.

Employed females in the medical system were more vulnerable to stress before the pandemic, comparing to males working in the same period and in the same field, at 10% significance level (Pearson’s chi-square statistic = 8.772, p-value<0.1). The employed females were also more vulnerable to stress during the COVID-19 pandemic, comparing to employed males in the medical system (Pearson’s chi-square statistic = 8.348, p-value<0.1). Non-employed female students from the Faculty of Midwifery and Nursing were more vulnerable comparing to male colleagues before the pandemic (Pearson’s chi-square statistic = 10.236, p-value<0.1), but no significant statistical differences were identified between the two groups at 10% significance level during the medical crisis.

Employed nursing students living in rural environment were more vulnerable to stress comparing to employed ones from urban environment before the pandemic (Pearson’s chi-square statistic = 4.578, p-value<0.1) and during the medical crisis (Pearson’s chi-square statistic = 6.348, p-value<0.1). Non-employed students from villages were more stressed out comparing to those from cities/towns before the pandemic (Pearson’s chi-square statistic = 7.223, p-value<0.1), but no significant statistical differences are identified between the two groups at 10% significance level during the pandemic.

There are no differences between employed and non-employed nursing students regarding stress vulnerability before and during the pandemic when characteristics like marital status and age categories are considered. For example, married nursing students working in the medical system did not experience more stress comparing to non-married ones and employed students before and during the pandemic. Married nursing students without employment were not more vulnerable to stress comparing to non-married colleagues without employment before and during the pandemic. Younger employed and non-employed students were not more vulnerable to stress comparing to older colleagues before and during the medical crisis.

Some relative frequences were computed in order to identify the most powerful stressor indicated by employed students during the pandemic and the most efficient copying strategy in this period. Most of the employed nursing students in the sample considered that the fear of getting sick with the new coronavirus is the most important stressor for them (42.5% of the employed students during the pandemic). Lower percentages were registered by the rest of the factors of stress: huge workload (35.83%), lack of experience in managing a pandemic (1.67%), miscommunication and social distance (11.67%), more patients, comparing to the period before pandemic (4.17%), mask wearing (2.5%), the use of new equipment purchased during the pandemic (0.83%), more frequent use of disinfectants (0.83%).

Some coping strategies at work are indicated during the pandemic and the respondents have been asked to choose the most efficient one for them: self-control, colleagues’ support, involving of hospital management, family’s support and spiritual dimension reflected from various perspectives as contact with nature, recognision of God’s power, use of prayers and holy books, spiritual improvement.

During the pandemic, 34.17% of the employed nursing students considered that self-control helped them in coping with stress. 27.5% of them admitted that spiritual dimension was essential in overcoming the stressful situations in this period. 20% of employed nursing students asserted that family’s support was essential during the pandemic. During the pandemic, 14.16% of the employed nursing students appreciated the colleagues’ support as useful in surpassing stressful situations at work, while 4.17% of them considered that medical unit management had the most important contribution in surpassing the stressful situations. Therefore, we can state that self- control and spirituality are important factors in reducing stress among employed nursing students in the analyzed sample during the pandemic. In Table 4, the results indicate significant differences regarding stress scores according to copying strategy for employed nursing students during the pandemic. Employed students considered self-control as being the most efficienct strategy in order to face stress. They have lower stress scores comparing to those promoting spiritual dimension, others’ support and management implication.

## Discussion

In the context of the current medical crisis, the aim of this work is to compare vulnerability to stress of nursing students from Bucharest considering their status on labour market and the changes regarding the levels of stress with respect to the pre-pandemic period.

The objectives of the research are related to: hypotheses concerning comparisons between students’ levels of stress (employed students and non-employed ones before and during the pandemic, employed students before and during the pandemic, non-employed nursing students in the pre-pandemic period and during it), the impact of demographic characteristics concerning vulnerability to stress, the identification of the most severe stressor and the most efficient strategy to cope with stress for employed nursing students during the pandemic.

During the COVID-19 pandemic, the employed nursing students working in the medical system were more vulnerable to stress comparing to their colleagues without a workplace. Schwartzman has also revealed that there is a higher stress in case of employed students comparing to non-employed ones during the pandemic [18]. In a previous study, Achterberg et al. (2021) has indicated that employed people have experienced a higher level of stress during the pandemic, comparing to non-employed ones [25].

If, before the epidemic, the employed students experienced a low vulnerability to stress, during this medical crisis, the average score indicates a medium vulnerability to stress. During the pandemic, a moderate level of stress among nursing students has also been registered on a sample of 662 students [2] in Turkey, and 427 nursing students [26] in India, but comparisons with pre-pandemic period have not been made in these studies.

Employed nursing students were more vulnerable to stress (medium vulnerability to stress) during the pandemic, comparing to employed students with low vulnerability to stress before the pandemic. Previously, Aguiar-Quintana et al. (2021) revealed that nurses were more vulnerable to stress during this medical crisis comparing to the pre-pandemic period [24]. During the pandemic, non-employed nursing students in our sample were more vulnerable to stress comparing to non-employed students before the pandemic, but the vulnerability to stress remained low.

During the pandemic, the higher level of stress for employed nursing students comparing to non-employed ones is in line with the conclusion of Sheroun et al. (2020) regarding the Indian nursing students [26].

Gender is important in managing stressful situations. Employed females in our sample were more vulnerable to stress comparing to males in both periods. Non-employed females experienced stress more than males only before the pandemic, while during the medical crisis significant differences were noticed between the two groups. Our results are in line with other studies that confirm the higher vulnerability to stress of women comparing to men [2, 5] and that might be explained by the fact that women have additional duties at home (unpaid domestic work, children’s care) and perform emotional activities at work, more than expected. The endocrine system of females is different from that of males and this aspect makes females more sensitive in various contexts, affecting them deeply, emotionally speaking. The lack of difference between males and females who were employed during the pandemic, according to stress scores, might be explained by less exposure of women to external threats because of the previous lockdown period and before the survey had been conducted in June 2020.

Environment had a significant impact on employed nursing students in both periods and non-employed ones before the pandemic. Employed students coming from villages were more vulberable to stress comparing to those coming from cities or towns, contrary to the study of Lambert and Lambert (2008) [7]. Our result might be explained by the fact that many nursing students from villages commute to cities and towns, especially to Bucharest, the capital of Romania, which provides many opportunities of working in hospitals, clinics and dispensaries. In Romania, on the other hand, the standard of living is lower for people living in the country comparing to those living in the urban areas and that constraint villagers more. During the pandemic, no differences were noticed among non-employed nursing students according to environment, due to online lectures that do not imply commute for villagers.

In our study, a particular attention was paid to factors of stress and copying strategies to stress for employed and non-employed nursing students during the pandemic. Besides the stressful nature of the nursing duties, the COVID-19 pandemic increased specific stress factors that were also considered in this research and were confirmed by previous studies: the fear of getting sick with COVID-19, miscommunication and social distance at workplace, mask wearing, more frequent use of disinfectants, and increasing of patients’ number [2, 27]. Almost half of the employed nursing students in the medical system claimed that fear of getting sick with COVID-19 is the most severe stressor as Zhang et al. (2020) revealed for Chinese nurses [1]. A lower percentage (35.83%) of nursing students working as nurses believed that huge workload represented the most severe stressor for them. This workload might be explained by the fact that nurses are forced to help patients in critical conditions, physically and sometimes, mentally. In Romania, media has asserted that, during the pandemic, the stress of medical personnel is increased by the higher number of patients and by the understaffed. The stressful situation of doctors caused by the pandemic will constraint the nurses more. Miscommunication and social distance were claimed by 11.67% of the employed nursing students and it is a stronger stressor among respondents comparing to the existence of more patients. Mask wearing, the lack of experience in managing a pandemic, the use of new equipment purchased during the pandemic and more frequent use of disinfectants are indicated by the fewest students as the most important stressor.

The lack of experience and professional knowledge in managing a pandemic situation of these proportions were considered the most stressful factors for nurses in this period by Mo et al. [3]. However, in our study, only 1.67% of the employed nursing students claimed that lack of experience in managing a pandemic represented the biggest challenge for them.

In many hospitals in Romania, new equipment has been received during the medical crisis, but that has required additional qualification. This aspect did not represent the most stressful challenge for most of the nurses in our sample.

During the pandemic, most of the employed nursing students considered self-control as the most efficient copying strategy to stress (34.17% of the employed students). Self-control strategy is associated with good health of nurses, increases immunity and makes them less vulnerable to coronavirus [9].

There are other studies confirming that self-control is an efficient method to cope with stress and it might refer to: positive thinking, physical exercise, recreation, tolerance, self-learning, self-reliance, compassion for the patients being in a more difficult situation, caution to avoid other stressful situations and prayer [10, 28]. All these coping strategies improve physical, psychological, and mental comfort of nurses. Self-control requires self-regulation which overlaps the coping strategies as regulation for stress, even if coping has a direct impact on environment and external factors [29].

Only 20% of the employed nursing students considered families as being essential in this pandemic in the study of Elloker (2003) [30]. The support of colleagues is important in controlling the nurses’ vulnerability to stress, for 14.16% nursing students during the pandemic. The most important strategy remains self-control [31], comparing to other studies where it is asserted that the support from other people is the most important way for nurses to cope with the professional stress [32, 33]. The higher stress levels revealed in our study during the pandemic emphasizes the need for increased self-control mechanisms.

During the pandemic, 27.5% of the nursing students surpassed the stresfull situations with the aid of spiritual support. This copying strategy is the second as importance in our study. More levels were identified for the spirituality acts: individual stage consisting of personal opinions, primary and secondary levels (recognizing the power of God), coping resources (contact with nature), coping behavior (telling prayers and reading holy books) and spiritual improvement [9]. The importance of spiritual values in nurses’ lives helped them to preserve their employment and face stressful situations with patience and pace as previous studies revealed for clinical nurses [28], for nurses from hospices [34] and for various categories of nurses [33].

During the pandemic, only 4.17% of the nursing students believed that medical unit management had the highest contribution in reducing the stress. This low percentage for this copying strategy with stress suggests that management of medical units should be improved.

### Practical implications of results

Our results have practical implications for strategies to cope with stress during the pandemic, for the management of medical units and for the higher education nursing. The pandemic context could not be stopped by nurses, but they should try to adapt themselves to new conditions and control their emotions and behaviors. Given this situation, besides self-control, spirituality could be also an important pillar for nursing students working in Bucharest.

In addition to the previous recommendations that strictly refer to nurses’ acts, medical centers should implement strong policies in order to promote an adequate physical and structural environment. At institutional level, work security, flexible scheduling, interactive management and labor relations, good communication practices should be implemented by the managers of the medical units. Investment is necessary in order to promote those policies that help nurses in preventing diseases and maintaining good health. In addition, educational interventions are necessary in order to inform nurses about the strategies to cope with stress, maintain good health and good balance between work and profession.

Specific strategies are necessary for students preparing themselves to work as nurses. Educational initiatives are the most important strategies for students to prepare themselves in order to face the pandemic challenges in their future work. They should also be motivated to practice this work since there is a shortage of personnel in this field in Romania. Faculties should adapt their educational programs in order to include web-based learning activities about coping with stress and ensuring personnel health and safety.

### Research limitations and implications

Apart from these relevant results, our research presents few limits that allow us to indicate future directions of study. First, there are other factors that influence the vulnerability to stress which were not taken into account when computing the stress scores using Miller and Smith scale and the list of stressors proposed to students during the pandemic (for example, the status of student in parralel with the workplace, the difficulties encountered in his/her own family, the kind of department where nursing student works, technological challenges, degree of confidence in COVID-19 vaccine). Therefore, in a future study, nursing students should be asked to indicate other particular factors of stress with a significant impact on them.

Second, this research only focused on a quantitative analysis of vulnerability to stress. Most of the studies from literature have focused on qualitative approaches in understanding the stressful circumstances for nurses, but this work limited itself to quantitative approach, because the number of nursing students is quite large and costs and time for individual interviews are higher. The results based on our quantitative approach are useful for the implementation of general policies in the Romanian medical system, but the consultation of a psychiatrist is necessary in case of people with a high level of stress who need specialized treatment.

Third, our strategies to cope with stress were designed only for employed nursing students and may not be relevant for non-employed students. Different strategies in order to face stressful situations should be applied depending on the status of nursing students on labour market (employed or non-employed ones). The individuals who already work as nurses might need different strategies to cope with stress comparing to nursing students who are preparing themselves to start working in this field. However, the current recommendations for employed nursing students during the pandemic could be useful for non-employed colleagues when they are employed as nurses.

Fourth, the sample of nursing students is representative for Bucharest, the capital of Romania, but not at national level. Therefore, the conclusions of this study could not be generalized to the entire population of nursing students in Romania. However, the research could be extended in order to include the other university centers with faculties of nursing. Comparisons between students coming from all these faculties are useful to verify whether the same stressors affect them the same manner and whether the same copying strategy was the most efficient.

## Conclusions

Workplace stress has become a more critical challenge in the labour market. Stress can affect hormonal balance and normal physiology of employed nursing students, thus, learning the stress coping strategies is essential in order to maintain good health, an acceptable life quality and well-being.

The aim of this research is to assess the vulnerability to stress of nursing students depending on their status on labour market before and during the COVID-19 pandemic. The results indicated that employed nursing students were more vulnerable to stress comparing to non-employed students before and during the medical crisis. In addition, the nursing students working in the medical system experienced medium vulnerability to stress during the pandemic, comparing to those working before the pandemic who experienced low vulnerability to stress.

Excepting the non-employed students before the pandemic, the females in the sample were more vulnerable to stress comparing to males because of their specific endocrine system, work involving emotions and other household duties, while the students in rural environment experienced stress more than those living in cities/towns because of commute and lower standard of living.

During the pandemic, most of the employed nursing students indicated that the fear of getting sick with COVID-19 was the most stressful factor for them, while most of them claimed self-control as being the best strategy for them to copy with stress.

In a future study, other factors impacting the vulnerability to stress will be considered: the increased use of Internet for communication, including online lectures and degree of confidence in COVID-19 vaccine. Apart from these factors of stress, other stressors should be considered in a future study. The department in which nursing students work might impact the vulnerability to stress. The nurses in emergency department might be more stressed out comparing to those in other departments. Aspects related to family should also be considered in another research: fear to get sick with the virus, unemployment, less time spent with family because of nursing duties etc.

The quantitative approach will be completed with interviews with each nursing student based on their agreement and university ethical approval. In addition, the interviews will help us to identify different strategies to cope with stress for employed nursing students and non-employed students. A future research might include data from other universities in the country with this specialization, comparisons between faculties being necessary.

## Supporting information

S1 File (DOCX)
