# Supplementary material for: How did the COVID-19 pandemic impact the stress vulnerability of employed and non-employed nursing students in Romania?
Source: PLoS One. 2022 Mar 4;17(3):e0264920. doi: 10.1371/journal.pone.0264920 (PMC8896692; doi:10.1371/journal.pone.0264920)
Supplement: S1 File — (DOCX) [file pone.0264920.s001.docx]

### Appendix 1: Description of scoring and questions in the survey

### The responses received points from 1 to 5 according to degree of vulnerability to stress. There are five categories to be considered with associated points (from 1 point to 5 points based on the response): 1 (almost always), 2 (often), 3 (sometimes), 4 (rarely), 5 (never). The score for each individual is computed as a sum of points for each question.

### The following categories of vulnerabilities to stress were considered based on the computed scores:

- 0-10 points – resistance to stress;
- 11-29 points – low vulnerability to stress;
- 30-49 points – medium vulnerability to stress;
- 50-74 points – high vulnerability to stress;
- 75-80 points – extreme vulnerability to stress.

### The questions are presented below:

### 1. I eat at least one balanced meal a day.

### 2. I sleep 7-8 hours/night at least 4 times / week.

### 3. I receive and give affection regularly.

### 4. I have at least one relative I can count on and who lives less than 80 km away.

### 5. I do exercise at least 2 times/week.

### 6. I smoke less than half a pack of cigarettes/day.

### 7. I drink less than 5 alcoholic drinks/week.

### 8. My body weight is adequate for my height.

### 9. My income is adequate for basic expenses.

### 10. I find my strength in my religious faith.

### 11. I am regularly involved in social activities.

### 12. I have a network of friends and acquaintances.

### 13. I have one / more friends I trust.

### 14. I am in good health (including sight, hearing, teeth).

### 15. I am able to talk openly about my feelings when I am upset or worried.

### 16. I usually talk to the family/people I live with about household issues (household activities, money, daily chores).

### 17. I do something funny at least once a week.

### 18. I am able to organize my time efficiently.

### 19. I drink less than 3 cups of coffee or cola / day.

### 20. I take a quiet break during the day.

### 21. Gender

### a) female

### b) male

### 22. Marital status:

### a) married;

### b) unmarried.

### 23. Age group:

### a) 18-25 years;

### b) 26-29 years;

### c) 30-39 years;

### d) 40-49 years;

### e) 50-65 years.

### 23. I live in:

### a) urban environment;

### b) rural environment.

### 24. Status on labour market:

### a) employed;

### b) non-employed.

### 25. I live:

### a) alone;

### b) on rent;

### c) with parents;

### d) in student dorm.

### The next questions are for students working as nurses during the pandemic.

### 21. During the pandemic, the most stressful work factor for me was:

### a) fear of getting sick with the new coronavirus;

### b) miscommunication and social distance;

### c) mask wearing;

### d) more patients comparing to the period before the pandemic;

### e) more frequent use of disinfectants;

### f) huge workload;

### g) lack of experience in managing a pandemic;

### e) the use of new equipment purchased during the pandemic.

### 22. The most efficient work coping strategy for me is:

### a) self-control;

### b) spiritual dimension (recognision of God’s power, contact with nature, telling prayers and reading holy books, spiritual improvement);

### c) other colleagues’ support;

### d) family’s support;

### e) support from medical unit management.

Appendix 2: Algorithm for assessing stress vulnerability

We will consider D as a fictional variable that allows us to establish the two groups of analysis. In this research, two fictional variables were considered: labour market status (1 for employed students, 0 for non-employed students) and the pandemic (1 for students who responded to the survey during the COVID-19 pandemic, 0 for students who responded to the survey before the COVID-19 pandemic).

### In this case, *i* is the index for an individual in the sample and the two groups are denoted by 0 and 1: employed students (denoted by 1 in the index of Y) and non-employed students (denoted by 0 in the index of Y), students responding to the survey before the COVID-19 pandemic (denoted by 0 in the index of Y) and students responding to the survey after the COVID-19 pandemic (denoted by 1 in the index of Y). For the variable of interest denoted by Y, the average treatment (coping strategies) effect (ATE) is computed as in (1):

$E\left( Y_{1i} \right)-E\left( Y_{0i} \right)=\frac{1}{N}\cdot\sum_{i=1}^{N} Y_{1i}-\frac{1}{N}\cdot\sum_{i=1}^{N} Y_{0i}=\frac{1}{N}\cdot\sum_{i=1}^{N} \left( Y_{1i}-Y_{0i} \right)=E(Y_{1i}-Y_{0i})$ (1)

E- expected value (average) of Y variable

The average treatment effect on the treated known as ATET is computed as in equation (2):

$E\left( Y_{1i}|D_{i}=1 \right)-E\left( Y_{0i}|D_{i}=1 \right)=\frac{1}{\sum_{i=1}^{N} D_{i}}\sum_{i=1}^{N} D_{i}Y_{1i}-\frac{1}{\sum_{i=1}^{N} D_{i}}\sum_{i=1}^{N} D_{i}Y_{0i}=\frac{1}{\sum_{i=1}^{N} D_{i}}\sum_{i=1}^{N} D_{i}{(Y}_{1i}-Y_{0i})=E(Y_{1i}-Y_{0i}|D_{i}=1)$ (2)

The average treatment effect on the control known as ATEC is shown in (3):

$E\left( Y_{1i}|D_{i}=0 \right)-E\left( Y_{0i}|D_{i}=0 \right)=E\left( Y_{1i}-Y_{0i}|D_{i}=0 \right)$ (3)

$\sum_{i=1}^{N} D_{i}$represents the number of treated units (number of students with a certain characteristic).

In case of treated, $D_{i}Y_{1i}=Y_{1i}$. For controls, $D_{i}Y_{1i}=0$ .

The propensity score acts like a summary score. If X is the vector of variables employed to estimate the propensity score and a group of control and a group of treated units present the same propensity score, there is the same repartition of X.

The nearest neighbour matching implies five steps, the result being represented by a data series of 2 x $N^{T}$ observations (T- number of treated units):

- Random assortment of treated elements;
- In the case of the first element i=1, the calculation of the absolute difference between propensity score for i and propensity scores of control units is made;
- For the minimum absolute difference the control unit is matched by i=1;
- Match control from the pool of possible control is deleted;
- The algorithm is repeated for i=1.
